# The Emerging Environmental and Public Health Problem of Electronic Waste in India

**DOI:** 10.5696/2156-9614-7.15.1

**Published:** 2017-09-07

**Authors:** Veenu Joon, Renu Shahrawat, Meena Kapahi

**Affiliations:** 1 Ministry of Environment Forest and Climate Change, Jor Bagh, New Delhi, India; 2 National Institute of Health and Family Welfare, Munirka, New Delhi, India; 3 Department of Chemistry, Manav Rachna University, Sector 43, Faridabad, India

**Keywords:** e-waste, environmental impact, health effects, Indian scenario

## Abstract

**Background.:**

Monumental progress has been made in the area of information and communication technology, leading to a tremendous increase in use of electronic equipment, especially computers and mobile phones. The expansion of production and consumption of electronic equipment along with its shorter life span has led to the generation of tremendous amounts of electronic waste (e-waste). In addition, there is a high level of trans-boundary movement of these devices as second-hand electronic equipment from developed countries, in the name of bridging the digital gap.

**Objectives.:**

This paper reviews e-waste produced in India, its sources, composition, current management practices and their environmental and health implications. Fixing responsibility for waste disposal on producers, establishment of formal recycling facilities, and strict enforcement of legislation on e-waste are some of the options to address this rapidly growing problem.

**Discussion.:**

The exponential growth in production and consumption of electronic equipment has resulted in a surge of e-waste generation. Many electronic items contain hazardous substances including lead, mercury and cadmium. Informal recycling or disposing of such items pose serious threat to human health and the environment.

**Conclusions.:**

Strict enforcement of waste disposal laws are needed along with the implementation of health assessment studies to mitigate inappropriate management of end-of-life electronic wastes in developing countries.

## Introduction

India has observed monumental progress in information and communication technology, leading to a tremendous increase in electronic equipment usage, especially of computers and mobile phones. The expansion of production and consumption of electronic equipment has been exponential over the last two decades.^1^ The useful life of this electronic equipment is becoming shorter due to frequent modifications and upgrades in software and rapid changes in equipment features and capabilities that usually do not support older equipment.^2^ This high rate of obsolescence in the industry has generated one of the fastest growing waste streams in the world consisting of various types of electronic items. Electronic waste (e-waste) or the term ‘waste electrical and electronic equipment’(WEEE) is used to describe old, end-of-life and discarded products including discarded refrigerators, mobile phones, computers, monitors, cathode ray tubes (CRTs), printed circuit board, compact discs, headphones, and white goods such as liquid crystal displays (LCD)/plasma televisions, etc. E-waste management poses a great challenge due to growing quantities of waste. E-waste is one of the most complex waste streams due to a wide variety of products including assembled or highly integrated systems.^3^,^4^ Due to the great variety of product models, the recovery of resources from e-waste is very challenging.^5^ The management of e-waste at the end, therefore, becomes a difficult task for existing solid waste and hazardous waste management structures, resulting in its handling by the informal sector employing very crude methods. In developing countries like India, e-waste units engage men, women and children for sorting and recovery of the materials without adopting protection and safeguards measures. This not only leads to contamination of the environment, but also poses a serious health threat to people engaged in this occupation as well as to the people living in the proximity of e-waste management sites.

This paper reviews e-waste produced in India, its sources, composition, current management practices and their environmental and health implications. Fixing responsibility for waste disposal on producers, establishment of formal recycling facilities, and strict enforcement of legislation on e-waste are some of the options to address this rapidly growing problem.

## Discussion

### Sources and Composition of E-waste in India

#### Quantum of E-waste and Major Contributors

Since the early 1990s, with the opening up of Indian markets to multinational companies due to globalization, the information technology industry has been witnessing a surge in the substitution of domestically produced hardware by imports. Consequently, e-waste generation in India is likely to increase nearly three-fold, making India the 5th largest producer of e-waste.^6^ According to this study, the Indian e-waste stream consists of computer equipment (about 70%) followed by telecommunication equipment (12%), electrical equipment (8%), medical equipment (7%) and household sector waste (remaining percent).^6^

According to an Associated Chambers of Commerce and Industry of India c-Kinetics study, India generates 1.85 million tons of e-waste every year. It accounts for 4% of global e-waste and 2.5% of global gross domestic product.^7^ India has emerged as the world's second largest mobile phone market with 1.03 billion subscribers. Nearly 25% of this ends up as e-waste annually. Generally, post-usage handling practices such as collection, separation, disassembling and recycling are performed manually in India.^8^ Approximately 95% of generated e-waste is managed by the unorganized sector and scrap dealers who dismantle the discarded products instead of recycling them.^9^ Only 5% of India's total e-waste finds its way to formal recycling units due to India's poor infrastructure and recycling framework. This leads to a waste of natural resources and damage to the environment and health of the people engaged in these activities. In India, about 400,000 to 500,000 child laborers between 10–15 years of age are involved in various e-waste activities.^6^ According to one study, by the year 2020, the amount of e-waste from old computers and discarded mobile phones is expected to increase 18 times compared to the year 2007.^10^

**Figure 1:**
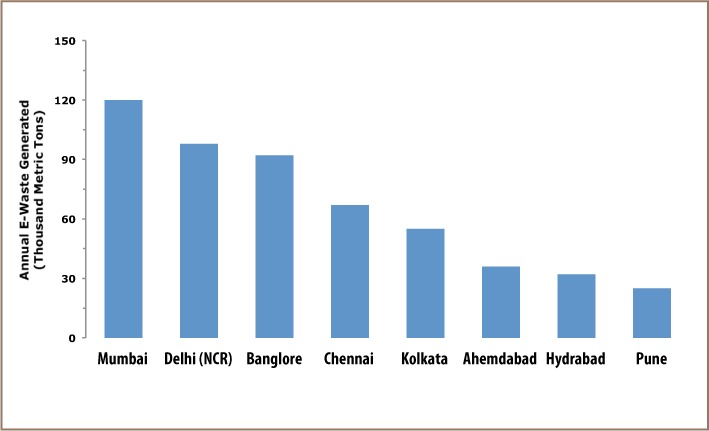
Major e-waste generating cities of India^13^

By sector, 71% of e-waste is generated by the government, public and private (industrial) sector, while individual households contribute about 16%.^11^ As per reports, the amount of e-waste generated differs by state in India. Among the 10 largest e-waste generating states, Maharashtra tops the list followed by Tamil Nadu, Andhra Pradesh, Uttar Pradesh, West Bengal, Delhi, Karnataka, Gujarat, Madhya Pradesh and Punjab.^12^ Similarly, there is a huge variation in e-waste generation in terms of cities, as only 65 cities of India generate more than 60% of total e-waste. Among the top ten cities generating e-waste, Mumbai ranks first, followed by Delhi, Bengaluru, Chennai, Kolkata, Ahmedabad, Hyderabad, Pune, Surat and Nagpur.^12^

**Abbreviations**
*CRTs*: Cathode ray tubes
*PCBs*: Polychlorinated biphenyls
*E-waste*: Electronic waste

#### E-waste Composition

The e-waste stream consists of more than 1000 different substances that fall into “hazardous” and “non-hazardous” categories. Broadly, it consists of ferrous (50%) and non-ferrous metals (13%), plastics (21%) and miscellaneous wastes.^14^ The hazardous elements category includes lead, mercury, arsenic, cadmium, selenium and hexavalent chromium and flame retardants.^14^ Table 1 shows different electronic waste components and their potential environmental hazards.

**Table 1:**
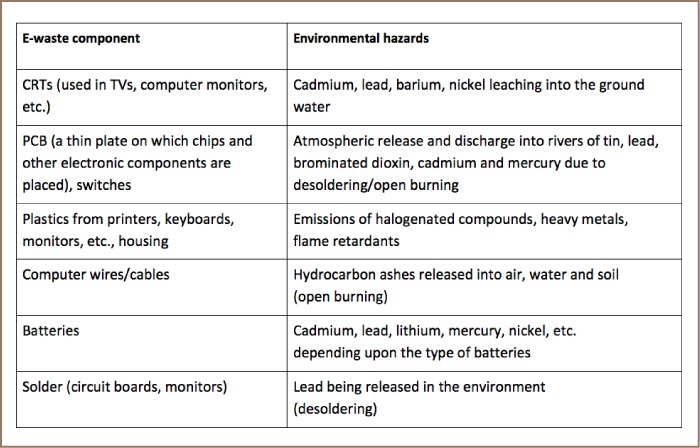
Different E-waste Components and Potential Environmental Hazards^14^,^43^

#### Import of E-waste

The import of e-waste is one of the major sources of e-waste in India. In developed countries, it is expensive to recycle discarded electronics. Thus, discarded electronic items (with shorter life spans) are exported to developing nations as charity or scrap in the name of bridging the digital gap with the developing world. Breivik et al. reported that 23% of e-waste was sent to non-OECD (Organization for Economic Co-operation and Development) countries by OECD countries in the year 2005.^15^ Therefore, countries like India, China and Pakistan experience the problems of e-waste not only from the domestic market, but also from unlawful imports from developed countries.^16^ Factors like the presence of cheap labor and absence of import regulations have supported the import of e-waste. However, it is illegal to import e-waste because India is a signatory to the Basel Convention for Trans-boundary Movement of Hazardous Substances. Used electronic products are being imported in a concealed manner or as reusable goods to be donated to educational institutions, making it difficult to estimate the quantities of e-waste imported into India. The goods are imported from countries which are not signatories to the convention, like the USA.^17^ Based on secondary sources, it is estimated that around 50,000 metric tons of e-waste is being imported into India every year.^18^ The Indian market represents a larger market than most high income countries.^19^ Indirect sources such as the uncontrolled import of obsolete e-products and improper recycling practices have led to the release of hazardous chemicals like polychlorinated biphenyls (PCBs) in these countries, in spite of the limited usage of these chemicals in the past.^20^

### Current Status of E-waste Management

#### E-waste Recycling Practices

The recycling of e-waste is a matter of huge concern. Only about 10% of e-waste is being recycled globally.^21^ India's capital alone produces about 67,000 metric tons of e-waste per year.^22^ The majority of this e-waste is being handled by untrained workers in slum areas without any personal protective equipment, posing a hazard to their health.^13^ The absence and/or poor implementation of legislation in India regarding the safe disposal of e-waste results in its unorganized collection, material segregation and extraction. Workers are likely to be exposed to varied levels of harmful elements released during different recycling operations via skin contact, ingestion or inhalation.^23^,^24^ Dismantling and sorting of one computer piece in the unorganized sector results in a very small return for workers, and exposes them to toxic fumes and hazardous chemicals.

These “backyard recyclers” lack exhaust-waste gas and wastewater treatment facilities and personal health protection equipment or safety devices. Workers engaged in formal recycling units are at a lower risk compared to workers in informal recycling units where environmental and occupational safety are neglected.^23^,^25^ According to one study, approximately 2/3 of these workers in India experienced breathing difficulties such as cough and choking.^6^ Higher concentrations of heavy metals like cobalt, chromium, lead and mercury have been reported in blood and urine samples of recycling workers engaged in formal recycling units as compared to the office workers in Sweden.^24^ High levels of copper, molybdenum, silver, cadmium, indium, antimony, thallium, and lead were observed in hair of male workers from e-waste recycling sites as compared to the reference workers in Bangalore and Chennai in India.^26^

Waste components that do not have any resale or reuse value are openly burnt or disposed of in open dumps.^27^ The situation is worsened due to the widespread nature of these recycling units. High concentrations of heavy metals in ground water, soil and plant samples have been found from Mandoli (Delhi), which harbors e-waste recycling units.^28^ High concentrations of PCBs in the metropolitan cities of India have been linked to proliferation of informal e-waste recycling units.^29^ In a study conducted in seven major Indian cities, maximum concentrations of PCBs were observed at two urban sites in Chennai city. The sites are situated close to the port (favoring e-waste import) and have informal e-waste processing units.^30^ Similarly, higher concentrations of PCBs have been reported in the soil at various locations in China and Pakistan.^31^,^32^

The owners running these small enterprises employ cheap and readily available labor. The labor force in the recycling sector has a low literacy rate and has very little awareness of the hazards of e-waste. Involvement of women and children in various recycling activities further aggravates the problem.^27^ Recycling and treatment facilities require a high initial investment and exceed recovered revenue, particularly those fitted with technologically advanced equipment and processes. However, e-waste recycling can be turned into a profitable venture if managed properly.^33^

Hence, there is an urgent need for an efficient and inexpensive recycling system to extract valuable materials with negligible environmental impacts. The recovered valuable materials can be used for refurbished electronic devices, saving manufacturing costs and providing employment opportunities.^34^ Torihara et al. have proposed the concept of a remote recycling system utilizing teleoperation technologies for small e-waste management units in Japan to save time and costs.^35^

#### Current Legislation in India

Many studies have revealed the danger posed by e-waste, its toxicity, carcinogenicity and other characteristics harmful to human health and the environment. This awareness has been the basis of global action leading to the tightening of laws and regulations. The Hazardous Wastes (Management, Handling and Trans-boundary Movement) Rules, 2008 regulate the export/import trade or trans-boundary movements of hazardous wastes, including e-waste.^36^

According to these rules, the import of hazardous wastes for disposal is not permitted. However, import of waste was permitted for reuse, recycling or reprocessing.^36^,^37^ This was followed by E-waste (Management & Handling Rules), 2011 which came into force in May 2012 with the clear objective of safeguarding the environment and promoting safe and efficient recycling of e-waste in India.^38^ The legislation brought e-waste collection, dismantling, recycling and disposal within the purview of the regulatory authorities with a clear set of responsibilities for all the stakeholders in the value chain. E-waste (Management) Rules, 2016, have recently been updated by the Ministry of Environment, Forest and Climate Change, Government of India vide Gazette Notification number G.S.R. 472(E), dated 23rd March, 2016.^39^

E-waste rules now include compact fluorescent lamps and other mercury containing lamps. The rules, for the first time, bring producers under extended producer responsibility. Producers were made responsible for the collection and exchange/hand over to authorized recyclers for disposal of e-waste in an eco-friendly manner. Penalties for the violation of these rules was also been introduced. After the new e-waste rules of 2016, producers have been made responsible for the collection of e-waste by authorized recyclers for disposal.^39^ As per the current data, approximately 178 recycling companies in operation are registered with the Central Pollution Control Board.^40^

With more emphasis on life-cycle accountability of products as per government rules, manufacturers now own a continued responsibility for their products after sale until final disposal (cradle-to-grave). This puts extra pressure on manufacturers to create take-back provisions and on designers to design recyclable and reusable materials. Designers and manufacturers therefore need to minimize the use of hazardous substances.^41^

### Environmental and Health Implications of E-waste

Many electronic items such as computers and mobile phones contain hazardous substances like lead, mercury and cadmium. Recycling or disposing of such items can pose serious threats to human health and the environment.^42^ When e-waste is disposed into landfills, these toxins can be released into the atmosphere or the surrounding land and lead to adverse environmental effects.

Populations are increasingly being exposed to hazardous chemicals. These hazardous chemicals are released during product manufacture and disposal. Some of these chemicals like heavy metals are toxic even in small amounts and can bioaccumulate in organisms. Some of these chemicals have the tendency of biomagnification, with their concentrations increasing at each trophic level along the food chain. Chemicals of high concern are known as persistent, bio-accumulative and toxic (PBTs) or persistent organic pollutants (POPs) that include organochlorine pesticides such as DDT (dichloro diphenyl trichloroethane), dioxins and furans.^44^ Improper handling and disposal of e-waste release PBTs and POPs can reach people thousands of miles away. Even small amounts of these extremely dangerous toxins can adversely affect human health. Potential health risks may result from direct contact with hazardous substances such as cadmium, lead, chromium, PCBs, as well as from other contaminated sources like water and soil.

Children are more vulnerable to the health risks of e-waste exposure and, therefore, need more protection. As they are still growing, children's intake of air, water and food in proportion to their weight is considerably increased compared to adults, resulting in greater risk of hazardous chemical absorption. In addition, since children's functional systems such as the central nervous, immune, reproductive and digestive system are still developing, exposure to toxic substances may be more harmful.^45^

Table 2 presents the harmful chemicals found in E-waste, their source and health effects on the human body.

**Table 2:**
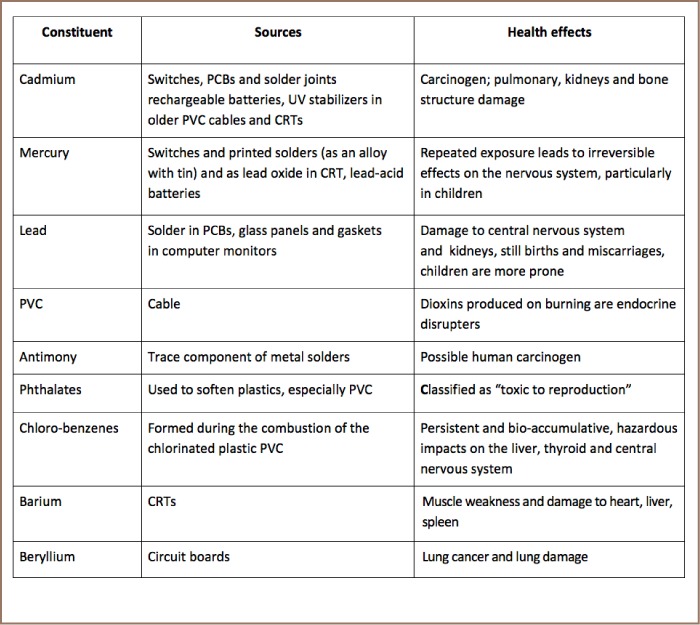
Chemicals Found in E-waste and Related Health Effects^14^,^23^,^25^

## Conclusions

India now joins the European Union, Japan, South Korea and some regions in the US and Canada who have all enacted legislation on extended producer responsibility to ensure that manufacturers are responsible for the re-use and recycling of their products at their end-of-life. This development clearly suggests a shift in e-waste flow from informal to formal recyclers and growth of a new clean e-waste management system in India. In addition, the government should further strengthen the following areas:

- Assessment of e-waste: Proper assessment of e-waste (along with the imports), its types and associated health hazards should be carried out.
- Encourage product re-design by asking electronic manufacturers to:
  a) Commit to product eco-design and target hazardous chemicals: Product designers should design products in such a way that they use no/negligible hazardous chemicals and can be easily disassembled into parts that can be reused, recycled or composted.
  b) Develop a corporate chemical policy: Policies should include the precautionary principle and an ongoing commitment to continually improve products with the safest chemicals and materials.
  c) Demand accountability throughout the supply chain: Requires suppliers to disclose the chemicals used in all components.
- Carry out health risk assessment studies: Health risk assessment studies are required for understanding the consequences of inappropriate management of end-of-life electronic wastes in developing countries.
- Increase consumer awareness: Initiatives to make consumers aware of ‘post-life’ handling of obsolete devices including take-back policies implemented by authorities.
- Training of workers: Training of workers involved in e-waste handling operations to avoid occupational health hazards is a prerequisite for safe e-waste management.
